# A 64 × 128 3D-Stacked SPAD Image Sensor for Low-Light Imaging

**DOI:** 10.3390/s24134358

**Published:** 2024-07-05

**Authors:** Zhe Wang, Xu Yang, Na Tian, Min Liu, Ziteng Cai, Peng Feng, Runjiang Dou, Shuangming Yu, Nanjian Wu, Jian Liu, Liyuan Liu

**Affiliations:** 1 State Key Laboratory of Superlattices and Microstructures, Institute of Semiconductors, Chinese Academy of Sciences, Beijing 100083, China; wangzhe@semi.ac.cn (Z.W.); yangxu@semi.ac.cn (X.Y.); tianna19@semi.ac.cn (N.T.); liumin@semi.ac.cn (M.L.); caiziteng@semi.ac.cn (Z.C.); fengpeng06@semi.ac.cn (P.F.); dourj@semi.ac.cn (R.D.); yushuangming@semi.ac.cn (S.Y.); nanjian@semi.ac.cn (N.W.); 2 College of Materials Science and Opto-Electronics Technology, University of Chinese Academy of Sciences, Beijing 100049, China; 3 School of Electronic, Electrical and Communication Engineering, University of Chinese Academy of Sciences, Beijing 100049, China

**Keywords:** SPAD, 3D stacking, single photon imaging, low-light imaging

## Abstract

Low-light imaging capabilities are in urgent demand in many fields, such as security surveillance, night-time autonomous driving, wilderness rescue, and environmental monitoring. The excellent performance of SPAD devices gives them significant potential for applications in low-light imaging. This article presents a 64 (rows) × 128 (columns) SPAD image sensor designed for low-light imaging. The chip utilizes a three-dimensional stacking architecture and microlens technology, combined with compact gated pixel circuits designed with thick-gate MOS transistors, which further enhance the SPAD’s photosensitivity. The configurable digital control circuit allows for the adjustment of exposure time, enabling the sensor to adapt to different lighting conditions. The chip exhibits very low dark noise levels, with an average DCR of 41.5 cps at 2.4 V excess bias voltage. Additionally, it employs a denoising algorithm specifically developed for the SPAD image sensor, achieving two-dimensional grayscale imaging under 6 × 10^−4^ lux illumination conditions, demonstrating excellent low-light imaging capabilities. The chip designed in this paper fully leverages the performance advantages of SPAD image sensors and holds promise for applications in various fields requiring low-light imaging capabilities.

## 1. Introduction

Image sensors play an important role in modern society. Single-photon imaging technology based on single-photon avalanche diodes (SPADs) is an emerging new visual technology. SPAD image sensors have single-photon sensitivity, high dynamic range (HDR), and picosecond time resolution [1,2,3]. Compared to CMOS image sensors, they can achieve various imaging functions such as HDR two-dimensional (2D) imaging [4,5,6], three-dimensional (3D) imaging [7,8,9,10], and fluorescence lifetime imaging (FLIM) [11,12,13]. Kumagai et al. designed a 3D SPAD sensor capable of measuring distances up to 150–200 m, with an accuracy of 0.15–0.3 m and a 3D imaging frame rate of 20 fps [7]. Ulku et al. designed a SPAD sensor known as “SwissSPAD2”, which has a resolution of 512 × 512, a fill factor of 10.5%, and a minimum gating time of 5.75 ns [12]. SPAD sensors can be applied in multiple fields including security surveillance, autonomous driving, and biomedicine. Their broad application prospects have prompted many companies and research institutions to conduct studies on this technology [14,15].

SPADs, combined with avalanche quenching circuits, can achieve fully digital readout, making them highly suitable for large-scale array integration. In recent years, with the rapid development of semiconductor process technology, using low-cost CMOS processes to achieve on-chip integration of SPAD arrays with large-scale mixed-signal circuits has become a research trend [16,17]. With the evolution of process nodes, the size of SPADs has gradually decreased [18], and the scale of the arrays has gradually increased. Morimoto et al. achieved the first megapixel SPAD image sensor based on a 180 nm CMOS process [19]. In 2016, Abbas et al. reported the first 3D stacking and backside-illuminated (BSI) SPAD sensor [20]. The introducing of 3D stacking and BSI technologies has further improved the integration of the sensors, enabling superior performance and complex functions [21]. This advancement is gradually transitioning SPAD sensors from scientific research to practical applications.

There has been a pressing demand for imagers with low-light imaging capabilities in many fields, such as security surveillance, night-time autonomous driving, wilderness rescue, and environmental monitoring. SPAD devices’ single-photon sensitivity endows them with significant potential applications in low-light imaging [22,23,24]. However, the noise level directly affects the capabilities of sensors. SPAD sensors do not require an additional analog-to-digital converter (ADC), thereby reducing the noise introduced by ADC. Meanwhile, SPADs have specific performance metrics such as photon detection probability (PDP), which reflects photosensitivity, and dark count rate (DCR), which reflects dark noise levels [25]. Large-scale SPAD arrays also face uniformity challenges caused by processing. As early as 2013, Bronzi et al. reported that in a 0.35 μm process node, about 30% of the 100 μm SPADs were classified as noisy devices [26]. In 2019, Zhang et al. designed a SPAD sensor based on a 180 nm CMOS process. Even with a median DCR of 195 cps, 6.2% of the SPADs still had a DCR greater than 1 k cps [9]. We have enhanced the photosensitivity of SPADs through chip architecture and circuit design, reduced noise interference, and mitigated pixel non-uniformity through an algorithm, thereby fully leveraging the excellent low-light imaging capabilities of SPAD sensors.

In this paper, a 64 (rows) × 128 (columns) SPAD sensor for low-light imaging is implemented. The chip adopts a 3D stacking architecture, which enhances the pixel fill factor. Additionally, it utilizes a compact gated pixel circuit designed with thick-gate MOS transistors, allowing for an expanded SPAD excess bias voltage and enhanced photon detection capabilities. The chip incorporates configurable digital control circuits, allowing the sensor to adapt to varying lighting conditions by configuring different exposure times. Moreover, it maintains very low levels of dark noise. Coupled with specific designed denoising algorithms, the chip can achieve 2D imaging under 6 × 10^−4^ lux illumination conditions. In Section 2 of this paper, we will introduce the chip architecture, circuit modules, and the denoising algorithm. Section 3 will discuss the testing system specifically designed for this chip and present the corresponding test results.

## 2. Methods

### 2.1. Chip Architecture

**Figure 1:**
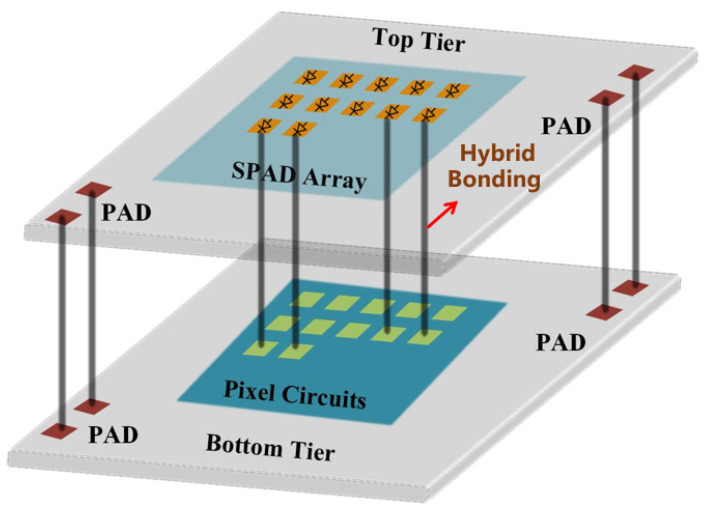
The 3D stacking chip architecture.

Figure 1 shows the architecture of the 3D stacking chip. The 64 (rows) × 128 (columns) SPAD array was implemented in the upper chip fabricated in a 180 nm process, while the pixel circuit array and other modules were implemented in the lower chip fabricated in a 130 nm process. The top-layer SPAD device array and the bottom-layer pixel circuit array are interconnected at the pixel level in the vertical direction through Cu-Cu hybrid bonding [27,28]. The I/O signals of the bottom-layer chip are also routed out from the top-layer chip through a combination of hybrid bonding and through silicon via (TSV).

Three-dimensional stacking architecture, which separates the device from the circuit, allows for the optimization of processes for the two layers independently [21,29]. In terms of the SPAD device structure, adjustments can be made without affecting the process of the underlying circuit chip, providing greater flexibility [30]. Three-dimensional stacking architecture is more conducive to integrating BSI SPAD devices [31]. Compared to front-side-illuminated (FSI) SPAD device structures, BSI structures reduce obstruction and losses caused by metal layers, thus enhancing photon detection probability. Simultaneously, placing the pixel circuitry on the bottom chip also helps to increase the SPAD array fill factor, enhance detection sensitivity, and further improve the sensor’s low-light imaging performance.

**Figure 2:**
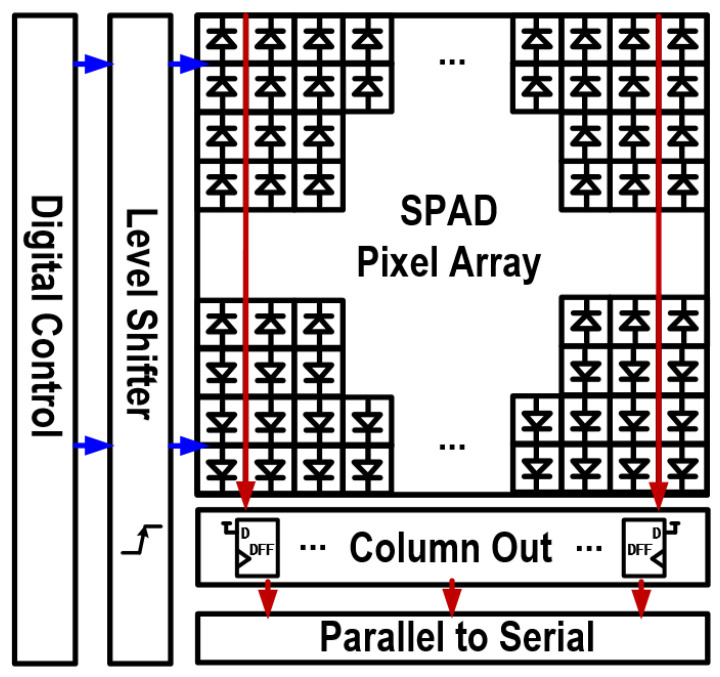
Block diagram of the sensor chip.

The chip circuit architecture, as shown in Figure 2, consists of the SPAD pixel array, readout circuit, serial conversion module, digital logic control circuit, and voltage conversion module. The SPAD pixel array consists of the SPAD array from the top chip and the pixel circuit from the bottom chip. The other circuit modules are all located in the bottom chip. A sought-after column-parallel readout circuit architecture in which each column shares one readout circuit is employed in this work [32,33]. Compared to pixel-level readout architectures, it can avoid increasing the complexity of pixel circuits. Unlike chip-level readout architectures, it can achieve higher readout efficiency, resulting in higher frame rates [34]. The chip operates in a rolling shutter mode, where control signals are generated by digital logic circuits to be sequentially exposed row by row in the pixel array. The exposure results are then captured by the readout circuitry, undergo parallel-to-serial conversion, and are finally outputted externally. Compared to global exposure, rolling shutter artifacts could appear when capturing fast-moving objects. A total of 16 I/O pads are used for data output, achieving a total readout rate of 960 Mbps with a 60 MHz system clock.

### 2.2. Pixel and Readout Circuits

**Figure 3:**
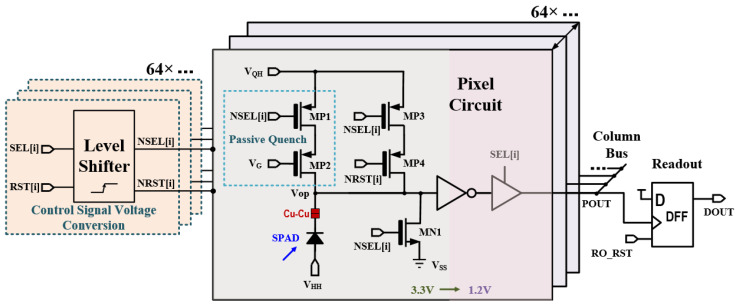
Pixel and readout circuit.

The pixel circuitry controls the activation/deactivation of SPAD devices as well as SPAD quenching, which occupies a critical core role in a single-photon image sensor [35]. Depending on the pixel operation mode, it can be divided into two types: free-running and gated [36]. In the free-running mode, the SPAD can trigger an avalanche as long as it is not in the dead time. In the gated mode, an external control signal is applied to the SPAD, periodically enabling or disabling it according to the sensor’s operating state. This reduces pixel power consumption and avoids unnecessary detection. A schematic of the gated pixel quenching circuit and readout circuit designed in this paper is shown in Figure 3. It is divided into 3.3 V and 1.2 V voltage domains. The part of the circuit directly connected to the SPAD uses 3.3 V MOS transistors, which, compared to 1.2 V MOS transistors, can increase the SPAD’s excess bias voltage range, improve the device’s photon detection capability, and thereby enhance low-light imaging performance. The other part uses 1.2 V MOS transistors to convert the avalanche signal and output it to the subsequent readout circuit.

The digital logic circuit also operates with a 1.2 V power supply. Therefore, we designed a specific voltage conversion module (level shifter) to buffer the 1.2 V control signals generated by the digital logic circuit to the 3.3 V pixel circuitry, as shown in Figure 3. Since the rolling shutter mode is used, each row can share one voltage conversion module. To simplify the pixel circuitry, the level shifter was arranged outside the pixel array, as shown in Figure 2.

The SPAD anode is connected to a negative voltage V_HH_ (around −20.5 V), placing the SPAD device in reverse bias, and the pixel circuitry is connected to the SPAD cathode V_OP_. SEL/NSEL is the selection signal, which controls the activation and deactivation of pixels. RST/NRST is the reset signal, which resets the pixel to its working state. When the pixel is deactivated, the MN1 transistor connects the SPAD cathode to ground, causing the voltage lower than the avalanche breakdown voltage (BV), thus putting the device in a non-operational state. When the pixel is opened and reset, the MP3 and MP4 transistor are opened, raising the voltage of the SPAD cathode to V_QH_, after which this channel is closed. At this point, the voltage across the SPAD is greater than BV, putting the device in a light-sensitive state. When triggered by a light signal, it will produce an avalanche signal. Meanwhile, the MP2 transistor controlled by the V_G_ signal acts as a variable resistor, enabling passive quenching of the SPAD device.

**Figure 4:**
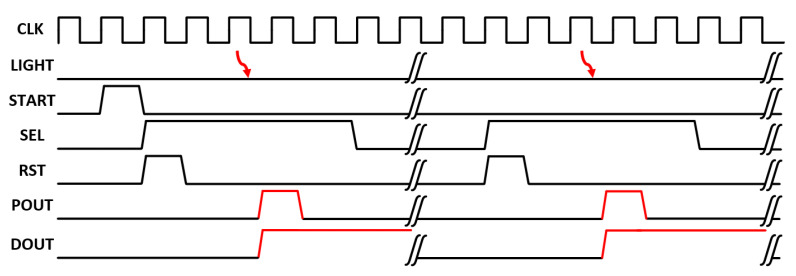
Timing diagram of pixel and readout circuit.

The timing diagram of the pixel circuitry and readout circuitry is shown in Figure 4. After the START signal initiates the chip, pixels are selected and reset by SEL and RST. If a light signal triggers avalanche in the SPAD, a falling-edge signal will be generated at V_OP_. This signal is then inverted by an inverter to generate a rising-edge pulse signal of 1.2 V. Subsequently, it passes through a three-state gate and is then output to the column bus POUT. The readout circuit employs a D flip-flop to sample and hold the electrical pulse signal output from the bus. The designed pixel circuit uses a minimal number of MOS transistors to achieve SPAD gating and quenching functions while enhancing the SPAD’s photon detection capability. This design lays a solid foundation for the sensor to achieve low-light imaging.

### 2.3. Digital Logic Control

A digital logic control circuit is designed to generate pixel and data readout control signals. This allows the pixel and readout circuit to work together to achieve rolling exposure imaging. The digital module can import configuration parameters from outside the chip. Different configurations can achieve various exposure times, row exposure intervals, and frame exposure intervals. By adopting a configurable approach, the sensor’s imaging frame rate can be easily regulated. Additionally, configuring different exposure times allows the sensor to adapt to varying lighting conditions. In low-light environments, the exposure time can be appropriately extended.

**Figure 5:**
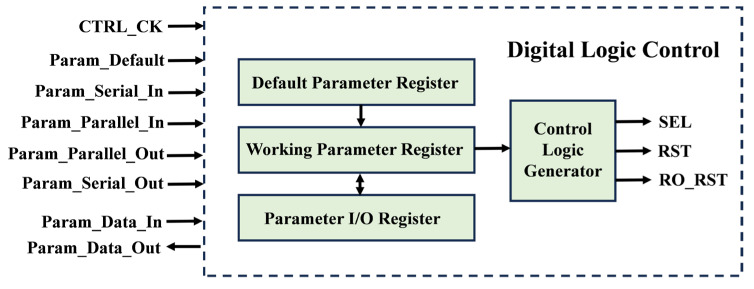
Schematic view of digital logic control circuit.

The structure of this module is shown in Figure 5. It includes three parameter registers: a default parameter register, a working parameter register, and a parameter input/output (I/O) register, as well as a control signal generator. The bit width of the three registers is 448 bits. The function of the default parameter register is to store a set of typical parameters, allowing the chip to operate normally without external input. The function of the working parameter register is to store the parameters used during the chip’s operation. The control signal generator will generate the control signals based on the working parameter register. The parameter I/O register serves as the interface between the module and the parameter data outside the chip.

**Figure 6:**
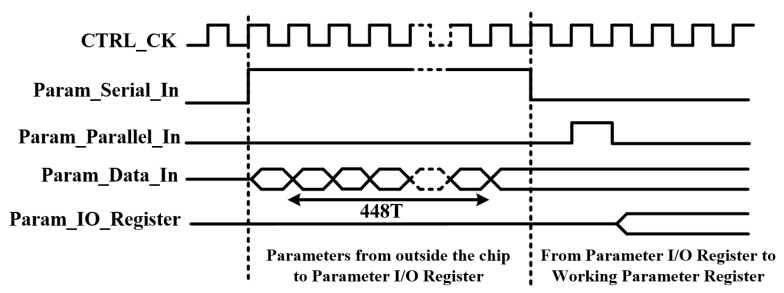
Timing diagram of parameter configuration for digital logic control circuit.

The operation of this digital control circuit under two modes will be discussed using both default parameters and externally input parameters. Upon receiving a pulse of the “Param_Default” signal, the chip will load the parameters stored in the default register into the working register. Subsequently, the chip will operate according to the configuration set by the default parameters. Figure 6 depicts the timing diagram when using externally input parameters. When the chip receives the “Param_Serial_In” signal, parameter data will be serially inputted into the I/O register, totaling 448 bits. Afterward, upon receiving the “Param_Parallel_In” signal, the parameter from I/O register will be loaded into the working register. Consequently, the chip will operate according to the configuration set by these parameters. The parameters used by the chip can also be outputted through the “Param_Data_Out” port to the exterior, thereby confirming the configuration employed by the chip.

### 2.4. Denoise Processing

**Figure 7:**
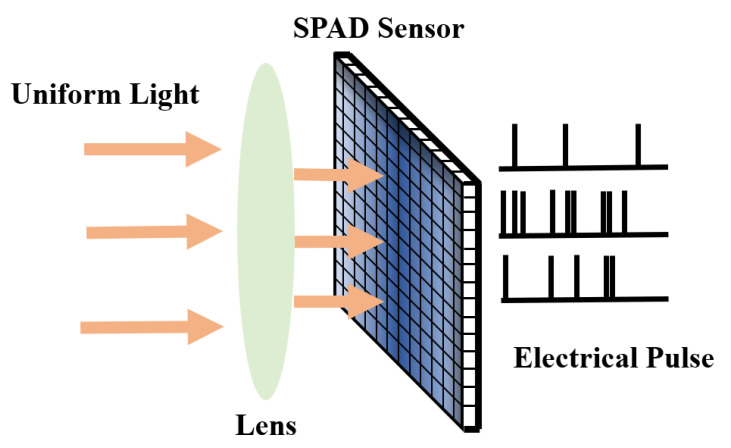
Diagram of photoelectric conversion based on SPAD sensor.

The principle of 2D imaging for SPAD sensors is to convert incident light into discrete electrical pulses, where the pulse density reflects the intensity of the light. Due to the nonideality of the manufacturing process of SPAD devices, different SPADs in the pixel array have varying PDP and DCR values. This results in different electrical pulse sequences even under completely uniform illumination conditions, as shown in Figure 7. In SPAD sensors, the pixel array directly outputs digital pulse signals, eliminating the need for further AD conversion. Therefore, the non-uniformity of the pixel array’s PDP and DCR contribute significant noise in SPAD image sensors.

The noise caused by the non-uniformity of the pixel array can worse the imaging results. Specific denoising algorithms is needed for post-processing to reduce this noise, thereby further enhancing the low-light imaging capability of SPAD sensor. This paper employs the modeling method from reference [37] to model the imaging process of the SPAD sensor. Based on this model, the imaging results are processed to achieve denoising. The principles of this algorithm are detailed below.

The output of a SPAD pixel can be represented as a pulse sequence:

(1) $$Z=\{z\left(1\right),\;z\left(2\right),\;z\left(3\right),\ldots z(i)\}$$

where $z\left(i\right)$ = 1 or 0. For a pixel (x,y), when a sufficient number of exposures, such as *M* times, are accumulated, the measured avalanche occurrence rate $A_{xy}$ will gradually approach the intrinsic avalanche probability $P_{xy}$ of the SPAD:

(2) $$A_{xy}=\frac{\sum_{M}z_{xy}(i)}{M}\sim P_{xy}$$

The intrinsic avalanche probability $P_{xy}$ of the device within a single exposure time can be expressed as

(3) $$P_{xy}=1-P_{nL,\;xy}P_{nD,xy}$$

where $P_{nL,\;xy}$ is the probability of not being triggered by incident light, and $P_{nD,xy}$ is the probability of not being triggered by dark noise. Because whether one incident photon triggers an avalanche is an independent event, $P_{nL,\;xy}$ can be further expressed as

(4) $$P_{nL,\;xy}={\left(1-{SP}_{xy}\right)}^{n(x,y)}$$

where ${SP}_{xy}\;$ is the PDP of SPAD pixel (x,y), n(x,y) is the total number of incident photons. Dark counts follow a Poisson distribution, so $P_{nD,xy}$ can be expressed as

(5) $$P_{nD,xy}=e^{-{SD}_{xy}\times T}$$

where ${\;SD}_{xy}\;$ is the DCR of SPAD pixel (x,y), T is the single exposure time. Substituting Equations (3) and (4) and approximating (5) with Taylor expansion into Equation (2) can obtain

(6) $$A_{xy}=\frac{\sum_{M}z_{xy}(i)}{M}=1-(1-{SD}_{xy}\times T){\left(1-{SP}_{xy}\right)}^{n(x,y)}$$

From there, we can derive the following equation:

(7) $$n(x,y)={log}_{1-{SP}_{xy}}\frac{M-\sum_{M}z_{xy}(i)}{M-{SD}_{xy}\times T\times M}$$

n(x,y) represents the ideal scenario of incident photons, while $\sum_{M}z_{xy}(i)$ represents the tested pixel output results. The model includes the influence of pixel PDP and DCR on the imaging. Therefore, by processing the original imaging results based on this algorithm, we can reduce the impact of noise caused by the non-uniformity of pixel device performance. This denoising process can fully leverage the performance advantages of SPAD devices, further enhancing the low-light imaging capabilities of SPAD sensors.

## 3. Results and Discussion

**Figure 8:**
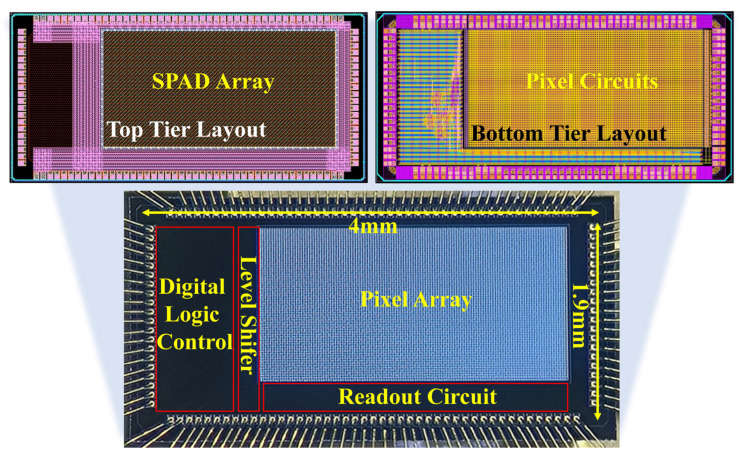
Layout and microscope of the chip.

Since the chip utilizes a 3D stacking architecture, an elaborate layout plan and de-sign are necessary for the multi-layer chip. The upper plane of Figure 8 depicts the layout and microscope of the SPAD sensor chip including the upper SPAD array chip and the lower circuit chip. The overall sizes of the upper and lower chip layouts remain completely identical, and the positions of the I/O pads also match strictly. Due to pixel-level signal connections, the unit sizes and positions of the pixel circuit array on the lower chip and the SPAD array on the upper chip should be completely identical, with each pixel corresponding to its counterpart. The individual size of the SPAD in this chip is 21 μm × 21 μm, as is the individual pixel circuit dimension. Within the constrained layout space, it is necessary to arrange the various components of the pixel circuit reasonably, considering both the convenience of overall wiring connections for the pixel array and the power supply capability.

The final chip photograph, after fabrication, is shown in the lower half of Figure 8. The overall size is 1.9 mm × 4 mm, with a 64 (rows) × 128 (columns) pixel array. The positions of the circuit modules such as the pixel array, digital control circuit, level shifter, and readout circuit are depicted in the chip photo. The chip features a layer of microlenses on the surface, which further enhance the pixel fill factor and improve the sensor’s low-light detection performance [38,39,40].

### 3.1. Measurement System

**Figure 9:**
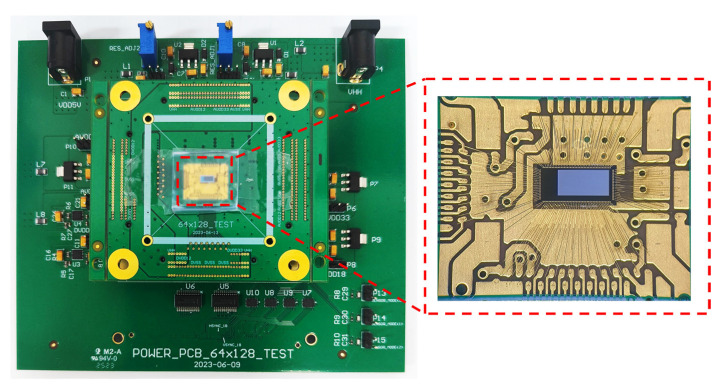
Photo of test board and chip wired to PCB.

Testing system is specifically designed for the chip. The photograph of PCB test board is shown in Figure 9. We adopt a split configuration with a main board and a daughter board, allowing for convenient replacement when testing different chips. The chip is directly wire-bonded to the daughter board, as shown on the right side of Figure 9, and all signals are routed to the main board. The main board supplies power to the chip. Since the FPGA signal voltage is not consistent with the chip’s I/O voltage, the main board also includes a voltage conversion module. Key signal and data lines have been designed with equal length in the PCB layout to prevent issues caused by transmission line delays.

**Figure 10:**
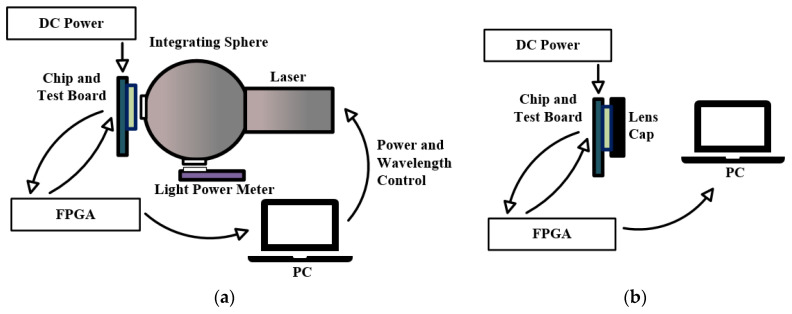
Schematic view of test system: (**a**) PDP test system; (**b**) DCR test system.

The pixel characteristics (PDP and DCR) testing system is shown in Figure 10. The PDP testing system consists of a DC power, chip and test board, light source and integrating sphere, light power meter, FPGA board, and a host computer, as shown in Figure 10a. The DC power provides power to the chip and test board. The light source and integrating sphere provide a uniform illumination condition. Additionally, we developed a dedicated FPGA testing project, which can configure the chip, receive output data, and transmit to the host computer.

By applying a specific power of light to the SPAD sensor and counting the digital output pulses, the PDP can be calculated [41]. For the PDP test, the laser power is first set and calibrated by the light power meter. The SPAD sensor is then placed at the output port of the integrating sphere (Quatek Inc., Shanghai, China). The light passes through the integrating sphere and uniformly illuminates the photosensitive area of the chip. The FPGA and host computer subsequently collect and analyze the output results. The DCR test system is shown in Figure 10b. The SPAD sensor is placed in a completely dark environment, and the DCR can be calculated based on the output data collected over a unit of time. The test results and calculation methods of PDP and DCR will be introduced in the next section.

**Figure 11:**
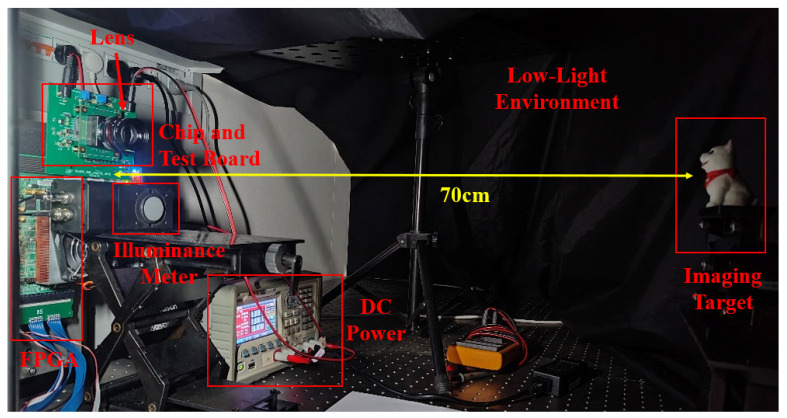
Low-light imaging experimental setup.

The specific built low-light imaging test system is shown in Figure 11. We equipped the chip with a suitable lens (F-number: 1.4) and placed the entire system in a darkroom. An adjustable light emitting diode (LED) light source was included to regulate the ambient light intensity, and a high-precision illuminance meter (Jinan FLS Optoelectronics Technology Co. Ltd., Jinan, China) was used to calibrate the ambient light intensity. The imaging target is a small dog figurine, positioned approximately 70 cm from the sensor. The chip’s low-light imaging results will be presented later.

### 3.2. Pixel Characteristics

For PDP test, to avoid photon accumulation and pile-up effects, the input light energy must ensure that the SPAD receives at most one photon per exposure cycle. This requires the incident light power to be extremely low. The incident light power density P(λ) needs to satisfy:

(8) $$P(\lambda )\times T\times A_{pixel}=\frac{hc}{\lambda }$$

where T is the single exposure time, $A_{pixel}$ is the photosensitive area of a single SPAD, h is Planck’s constant, c is the speed of light, and λ is the wavelength of the incident light.

Before testing, the light power meter must be used to measure and adjust the light source’s power to meet the above conditions. Different wavelengths require different power settings. Then, the PDP of SPAD (SP) can be calculated using the following formula:

(9) $$SP=\frac{N_{pixel}-N_{dark}}{M}$$

where M is the total number of exposures, $N_{pixel}$ is the number of times the pixel avalanched, and $N_{dark}$ is the number of outputs under dark conditions.

**Figure 12:**
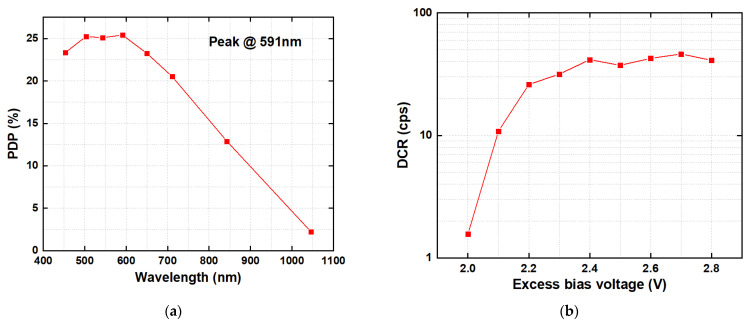
Measurement results of pixel characteristics: (**a**) PDP versus light wavelength at 2.4 V excess bias voltage; (**b**) DCR versus excess bias voltage at room temperature.

Figure 12a shows wavelength dependence of the pixel PDP at 2.4 V excess bias voltage, as obtained from the test. From the figure, it is evident that within the wavelength range of 453 nm to 1046 nm, the PDP initially increases with the wavelength, reaching a peak value of 25.4% at 591 nm. As the wavelength continues to increase, the PDP decreases, reaching 12.9% at 843 nm. Within the wavelength range of 453 nm to 711 nm, the pixel PDP remains above 20%.

For DCR, as mentioned before, the probability of no avalanche triggering in the exposure time for the SPAD is given by:

(10) $$P=e^{-SD\cdot T}$$

where SD is the DCR of the SPAD. While the statistically obtained probability of avalanche triggering for the SPAD within the exposure time T is:

(11) $$1-P=\frac{N_{dark}}{M}$$

According to Equations (10) and (11), the DCR of the SPAD can be calculated as:

(12) $$SD=-\frac{log(1-N_{dark}/M)}{T}$$

Figure 12b shows the variation of the SPAD mean DCR with excess bias voltage obtained at room temperature. It can be observed that the mean DCR increases with excess bias voltage when it is less than 2.4 V. Subsequently, between 2.4 V and 2.8 V, DCR exhibits only minor fluctuations. The DCR mainly originates from thermal noise, trap-assisted noise, and tunneling noise. As the excess bias voltage increases, tunneling noise will increase. However, the SPAD has a small proportion of tunneling noise due to a relatively thick depletion region. When the excess bias voltage exceeds 2.4 V, the impacts of other noise become more apparent, so the DCR tends to stabilize with further increases in voltage. At an excess bias voltage of 2.4 V, a mean pixel DCR is 41.5 cps. The excellent dark count level of the pixel lays a solid foundation for low-light imaging of the sensor.

### 3.3. Low-Light Imaging

**Figure 13:**
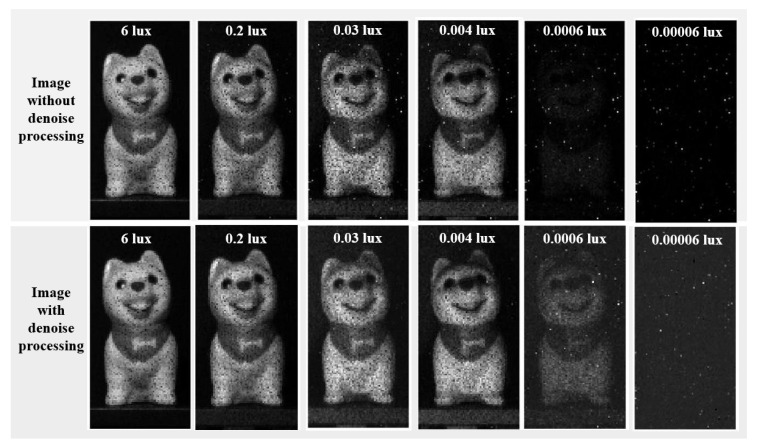
Imaging results under different illuminance with/without denoise processing.

The tested imaging results under different lighting conditions are shown in Figure 13. A 60 MHz system clock is used. The exposure time refers to the duration for which the SPAD pixels are activated. Depending on the parameter configurations, different exposure times can be achieved. To acquire better low-light imaging results, a SPAD pixel single exposure time of 233 ns is set. Additionally, 65,000 exposures are accumulated for one SPAD, with a total exposure time of 15 ms. The output pixel rate is 447 Mpixels/s with a 60 MHz system clock and a 233 ns exposure time.

In the upper part of Figure 13, the raw images without any data processing are displayed. Under illumination conditions greater than 4 × 10^−3^ lux, the imaging target is clearly visible. Under illumination conditions of 6 × 10^−4^ lux, the imaging target is faintly visible. However, under illumination conditions of 6 × 10^−5^ lux, the imaging target is completely invisible.

The lower part of Figure 13 shows the imaging results processed with the denoising algorithm detailed in Section 2.4. Under illumination conditions greater than 4 × 10^−3^ lux, as the original imaging results are already quite clear, the improvement is not very noticeable. However, under illumination conditions of 6 × 10^−4^ lux, the denoising process makes the previously blurry imaging results much clearer. The peak signal-to-noise ratio (PSNR), which is one of the metrics of image quality, is used to quantify the image quality under 6 × 10^−4^ lux. Using the image under 6 lux as the reference, the PSNR of the image under 6 × 10^−4^ lux is 10.8 dB without denoising. After applying the denoising algorithm, the PSNR improved to 12.5 dB. This improvement further validates the effectiveness of our denoising algorithm under low-light conditions. Under illumination conditions of 6 × 10^−5^ lux, the denoising process did not reveal any imaging targets due to the loss of target image information.

The SPAD image sensor designed in this paper can achieve 2D grayscale imaging under illumination conditions of 6 × 10^−4^ lux, effectively expanding the sensor’s application scenarios. To further expand the sensor’s limits and achieve imaging under illumination conditions of 10^−5^ lux or even lower, the sensor needs to have higher PDP and lower DCR levels.

**Figure 14:**
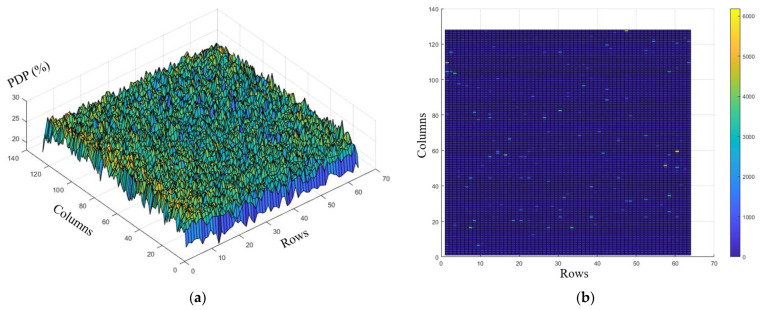
Sensor pixel array PDP and DCR maps: (**a**) pixel array PDP map at 591 nm; (**b**) pixel array DCR map at an excess bias voltage of 2.4 V.

**Figure 15:**
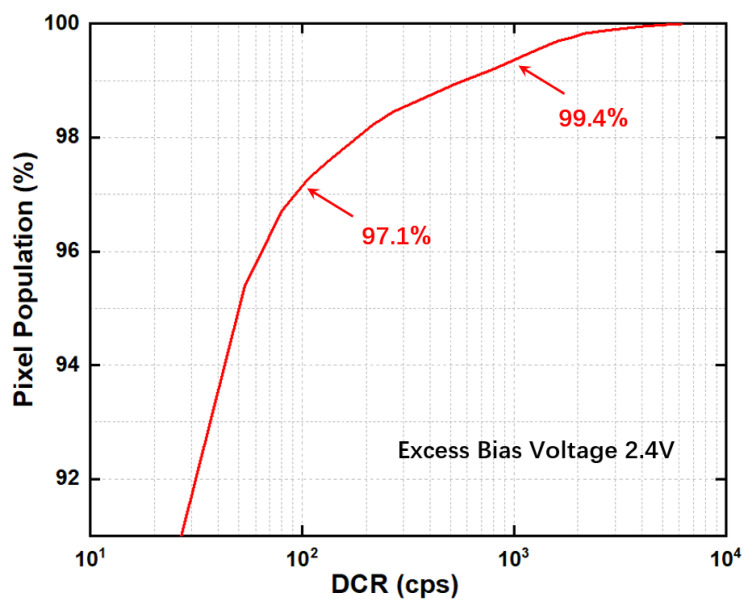
DCR population density at an excess bias voltage of 2.4 V.

Figure 14 shows the PDP and DCR map used in the above denoising algorithm. In Figure 14a, the PDP map of the chip pixel array at a wavelength of 591 nm is displayed, which reflects the uneven sensitivity of the chip. The average PDP of the pixel array is 25.4%, with a standard deviation of 1.4%. Figure 14b shows the DCR map of the pixel array at room temperature with an excess bias voltage of 2.4 V. The bright spots represent pixels with higher dark count levels. Figure 15 quantitatively shows the data distribution of the DCR map. In the entire pixel array, the number of pixels with a DCR less than 100 accounts for 97.1%, the number of pixels with a DCR between 100 and 1000 accounts for 2.3%, and the number of pixels with a DCR greater than 1000 accounts for 0.6%. The unevenness of both PDP and DCR can affect the final imaging results.

### 3.4. Comparison with Previous Chips

Conventional CMOS image sensors can also achieve low-light imaging capabilities by optimizing the pixels. The advantage of this approach is that the pixel size can be made very small. However, the challenges include increasing the pixel conversion gain while reducing readout noise. Conventional CMOS image sensors usually have more complex circuits, including analog-to-digital converters (ADCs), which result in various noise sources. In 2021, Ma et al. developed a quantum image sensor (QIS) based on 45 nm/65 nm 3D-stacked BSI CIS technology and demonstrated imaging results at 0.01 lux using an f/1.4 lens and an integration time of 600 ms [42]. The advantage of SPAD sensors is the use of avalanche effects, which provide higher gain and simpler circuit structures. Additionally, the primary source of noise only comes from the SPAD device itself. However, the current drawback of SPAD sensors is their relatively large pixel size. Both approaches require continuous technological advancements to achieve better low-light imaging capabilities.

In recent years, SPAD image sensors have become a research hotspot, with numerous SPAD-related studies being conducted across different fields. We selected chips that used 180 nm technology for SPAD array fabrication for comparison. The detailed comparison results are shown in Table 1. The pixel array size of the chip in this paper is slightly larger compared to the other three studies. PDP is a parameter independent of the fill factor, reflecting the inherent photosensitivity of the device. Photon detection efficiency (PDE) introduces the influence of the fill factor, taking the geometric factors of the device and the area of the pixel circuit into account. Due to the use of a 3D stacking architecture, the chip in this paper has a higher pixel fill factor and smaller pixel size. The peak PDP of the chip is not very high, but thanks to the higher fill factor and presence of microlens, a relatively good PDE can be achieved. Crucially, the chip has a very low DCR, which enables excellent low-light imaging performance. Taking the SPAD active area into account, the DCR density in reference [43] is 0.49 cps/μm^2^. In our work, the DCR normalized by the SPAD active area is 0.25 cps/μm^2^. The order of magnitude is similar, but numerically, the value in reference [43] is nearly twice ours. Most studies do not demonstrate the low-light imaging capability of chips under extreme conditions. Reference [44] provides some insights into this aspect. Due to its focus on imaging speed, it only shows the imaging results at an illumination of 0.5 lux with an integration time of 8.5 μs, and the DCR reported in reference [44] is an order of magnitude higher than ours.

## 4. Conclusions

This article introduces a 64 (rows) × 128 (columns) SPAD image sensor fabricated by 3D stacking technology, with a pixel size of 21 μm × 21 μm and a pixel array fill factor of 38%. The chip employs a compact gated pixel circuit designed with thick-gate MOS transistors, which enhance the SPAD excess bias voltage and improve photon detection capabilities. It features configurable digital control circuits, enabling the sensor to adapt to varying lighting conditions by configuring different exposure times. The chip has a very low level of dark noise, with an average DCR of 41.5 cps at a 2.4 V excess bias voltage. When combined with the denoising algorithm developed specifically for the SPAD sensor, it further extends the sensor’s low-light imaging capabilities. Our results indicate that this chip can achieve 2D imaging under 6 × 10^−4^ lux illumination conditions. It shows promise for applications in fields requiring low-light imaging, such as security surveillance and night-time autonomous driving.

## Figures and Tables

**Table 1:** Comparison of this work and previous research.

	Unit	This Work	[44]	[45]	[43]
Technology	-	180 nm/130 nm Stacked BSI	180 nm HV FSI	180 nm HV FSI	180 nm CIS FSI
Array Size	-	64 × 128	32 × 64	32 × 32	32 × 32
Pixel Size	μm	21 × 21	48 × 61	60 × 60	28.5 × 28.5
Microlens	-	Yes	/	/	/
Fill Factor *	%	38	13.4	7.2	28
Peak PDP	%	25.4	57	31	47.8
Median DCR	cps	41.5	810	1200	113
Low-Light Imaging	lux	10^−4^	10^−1^ **	NA	NA

* Without a microlens, not effective fill factor with microlens; ** with an integration time of 8.5 μs.

## Data Availability

Data are contained within the article.
